# Stream ichthyofauna of the Tapajós National Forest, Pará, Brazil

**DOI:** 10.3897/zookeys.580.6659

**Published:** 2016-04-12

**Authors:** Cárlison Silva-Oliveira, André Luiz Colares Canto, Frank Raynner Vasconcelos Ribeiro

**Affiliations:** 1 Programa de Pós-Graduação em Recursos Aquáticos Continentais Amazônicos, Instituto de Ciências e Tecnologia das Águas, Universidade Federal do Oeste do Pará. Rua Vera Paz, s/n 68035-110 Santarém, Pará, Brazil; 2 Universidade Federal do Oeste do Pará, Curso de Bacharelado em Ciências Biológicas. Campus Amazônia, sala 232. Avenida Mendonça Furtado, 2946, 68040-470 Santarém, Pará, Brazil

**Keywords:** Amazon, conservation, fish, Neotropical region, Tapajós River

## Abstract

The fish fauna of freshwater streams in the Tapajos National Forest was surveyed and a list of species is presented. The sampling was conducted from 2012 to 2013 during the dry season. Fish were collected with dip nets and seine nets in 22 streams of 1^st^ to 3^rd^ order. Sampling resulted in 3035 specimens belonging to 117 species, 27 families and six orders. The most abundant species were Bryconops aff. melanurus, *Hemigrammus belottii*, and *Hemigrammus analis*. Four undescribed species were recognized, one of which is known only from the area of this study. A significant dissimilarity was observed in fish species composition among drainage systems. This is the first survey of the stream ichthyofauna in the Tapajós National Forest, and it presents relevant information for future studies and decision-making in the management and conservation of fish fauna in this conservation unit.

## Abstract (pt)

A ictiofauna de riachos na Floresta Nacional do Tapajós foi inventariada e uma lista de espécies é apresentada. As amostragens foram realizadas de 2012 a 2013 durante o período de águas baixas. Os peixes foram coletados com redes de arrasto e peneiras em 22 riachos de 1^ª^ a 3^ª^ ordem. As amostragens resultaram em 3035 espécimes pertencentes a 117 espécies, 27 famílias e seis ordens. As espécies mais abundantes foram Bryconops aff. melanurus, *Hemigrammus belottii* and *Hemigrammus analis*. Quatro espécies novas foram reconhecidas, sendo uma conhecida apenas da área de estudo até o presente momento. Foi observada uma dissimilaridade significativa na composição de espécies de peixes entre os sistemas de drenagem. Esse é o primeiro inventário da ictiofauna de riachos na Floresta Nacional do Tapajós e apresenta informações relevantes para subsidiar estudos futuros e a tomada de decisões no gerenciamento da ictiofauna nessa Unidade de Conservação.

## Introduction

The Neotropical region has the richest and most diverse fauna of freshwater fishes in the world, reaching a number of more than 5400 valid species (Reis 2013) and estimates of the final number of more than 8000 species (Reis et al. 2016). Among its watersheds, the highest species richness is located in the Amazon River basin (Santos and Ferreira 1999; Reis et al. 2003), where the number of fish species remains undefined, particularly those inhabiting small streams. In these environments, despite having low primary production (Walker 1990), a rich fish fauna is supported, composed mainly of small-sized fish species (Henderson and Walker 1986; Castro 1999).

Several studies have contributed to our knowledge of the Neotropical fish fauna in recent years. Most noteworthy are those aimed at surveying the ichthyofauna (e.g. De Oliveira et al. 2009; Barros et al. 2011; Raiol et al. 2012; Pedroza et al. 2012), studies with focus on ecology that have tested the influence of environmental factors on the assemblage structure (e.g. Mendonça et al. 2005; Espirito-Santo et al. 2008; Dias et al. 2009), studies on natural history (e.g. Zuanon and Sazina 2004; Zuanon et al. 2006), feeding ecology (e.g. Gonçalves et al. 2013), new distribution records (e.g. Dagosta et al. 2012), and descriptions of new species (e.g. Kullander and Ferreira 2005; Lima et al. 2009; Sousa et al. 2010; Ribeiro et al. 2011; Dutra et al. 2012; Teixeira et al. 2013; Espíndola et al. 2014; Román-Valencia et al. 2014; Silva-Oliveira et al. 2015).

The main objective of the Tapajós National Forest (FLONA Tapajós), founded in 1974, has focused on the multiple use of forest resources and scientific research (SNUC 2000). However, studies of the fish fauna in streams are still needed. Collecting data on species composition in restricted geographical areas, such as conservation units, is an important initial step in decision-making related to the management of fish communities and conservation. Thus, the present study aimed to provide a list of fish species and to test difference in fish species composition among different drainage systems in the Tapajós National Forest.

## Materials and methods

### Study area

**Figure 1.**
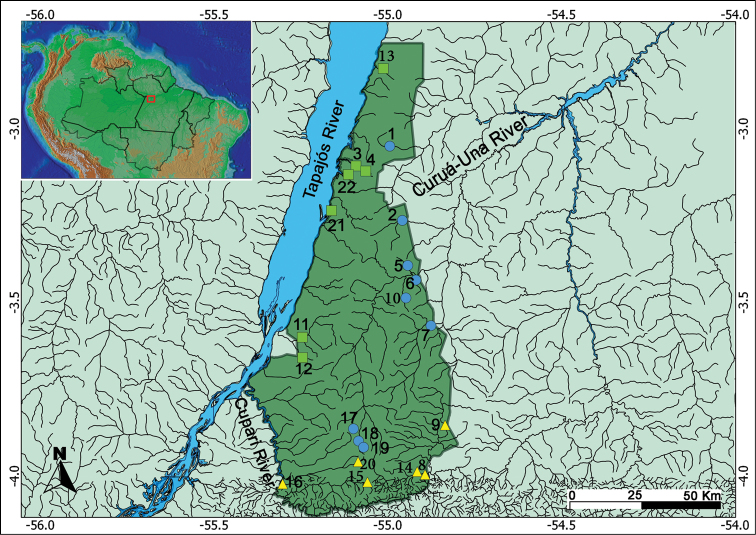
Map of the study area showing the collecting stations in drainage systems in the Tapajós National Forest, Pará State, Brazil. Green squares represent streams draining directly into the Tapajós River; blue dots represent streams draining into the Curuá-Una River, and yellow triangles represent streams draining into the Cupari River.

The Tapajós National Forest (FLONA Tapajós), located in western Pará State, approximately 3°24'S, 55°03'W (Fig. 1), holds an area over 527,000 hectares encompassing part of the Aveiro, Belterra, Placas, and Rurópolis municipalities (ICMBio 2014). The FLONA Tapajós is bordered in the west by the Tapajós River, in the east by the highway BR-163, connecting Cuiabá (Mato Grosso State) to Santarém (Pará State), in the south by the Cupari River, and in the north its border is perpendicular to intersection 65 km on BR 163 North. Streams in the FLONA Tapajós streams flow directly in the Tapajós River or drain into two distinct river systems − Curuá-Una and Cupari rivers.

### Data collection

**Figure 2.**
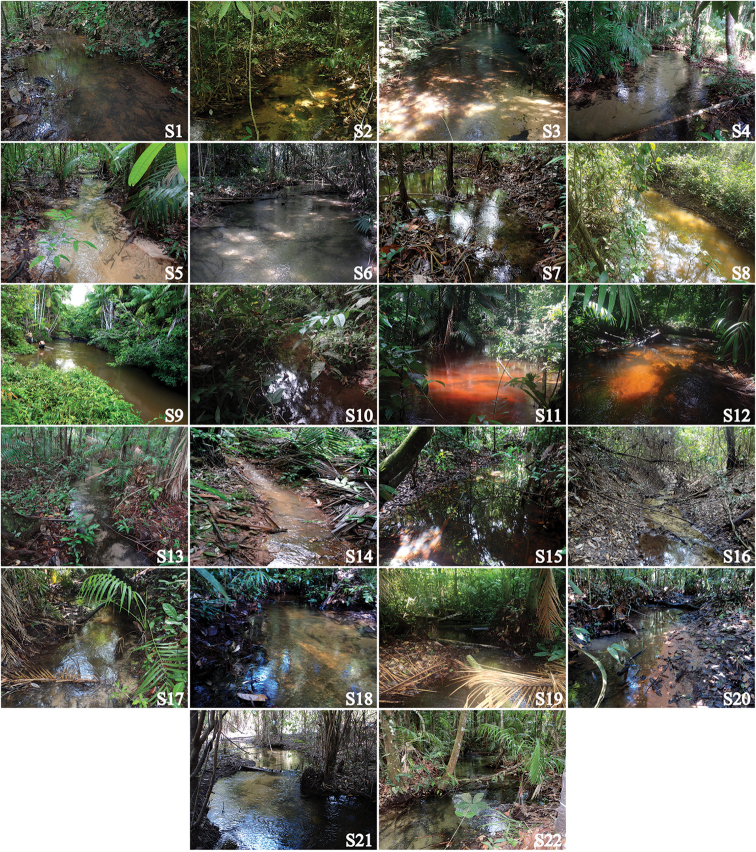
Sampled streams in the Tapajós National Forest, Pará State, Brazil.

Twenty-two streams of 1^st^ to 3^rd^ order were sampled (Fig. 2) during the dry season from September 2012 to November 2013. Nine streams belong to the Curuá-Una river system, six drain into the Cupari River, and seven flow directly in the Tapajós River (Table 1). Fish sampling followed a part of the protocol proposed by Mendonça et al. (2005), in which a 50-m section of each sampled stream was blocked with fine-mesh nets (5 mm between opposite knots). After blocking a section, two collectors were actively sampling for about two hours using dip nets and seine nets.

**Table 1.** Location of sampling stations in the Tapajós National Forest, Pará State, Brazil.

Station	Drainage	Locality	Geographical coordinates
**1**	Curuá-Una River	km 85 stream	03°02'50.9"S, 54°59'32.9"W
**2**	Curuá-Una River	unnamed stream	03°15'39.2"S, 54°57'22.7"W
**3**	Tapajós River	Corredor ecológico stream	03°15'39.2"S, 54°57'22.7"W
**4**	Tapajós River	unnamed stream	03°07'8.54"S, 55°03'42.4"W
**5**	Curuá-Una River	km 117 stream	03°23'26.2"S, 54°56'26.7"W
**6**	Curuá-Una River	unnamed stream	03°25'57.0"S, 54°55'01.8"W
**7**	Curuá-Una River	Onça stream	03°33'48.9"S, 54°52'26.3"W
**8**	Cupari River	Água preta stream	03°59'34.5"S, 54°53'27.5"W
**9**	Cupari River	unnamed stream	03°51'03.7"S, 54°50'00.0"W
**10**	Curuá-Una River	unnamed stream	03°29'02.1"S, 54°56'45.8"W
**11**	Tapajós River	Açu stream	03°35'49.4"S, 55°14'39.6"W
**12**	Tapajós River	Cachoeirinha stream	03°39'19.7"S, 55°14'37.1"W
**13**	Tapajós River	Maguari stream	02°49'26.9"S, 55°00'40.6"W
**14**	Cupari River	unnamed stream	03°59'04.3"S, 54°54'49.4"W
**15**	Cupari River	unnamed stream	04°00'52.5"S, 55°03'24.1"W
**16**	Cupari river River	unnamed stream	04°01'11.6"S, 55°18'02.7"W
**17**	Curuá-Una River	unnamed stream	03°51'41.7"S, 55°05'49.7"W
**18**	Curuá-Una River	unnamed stream	03°53'47.6"S, 55°04'56.7"W
**19**	Curuá-Una River	unnamed stream	03°54'53.3"S, 55°04'04.6"W
**20**	Cupari River	unnamed stream	03°57'21.5"S, 55°05'01.2"W
**21**	Tapajós River	unnamed stream	03°13'57.8"S, 55°09'36.9"W
**22**	Tapajós River	unnamed stream	03°07'44.8"S, 55°06'42.6"W

Specimens were anesthetized in a solution containing eugenol (clove oil), fixed in 10% formalin solution, and subsequently transferred to 70% ethanol. They were counted and identified to the lowest possible taxonomic level. Species were identified with the use of dichotomous keys for different taxonomic groups (e.g. Géry 1977; Kullander 1986; Vari 1992; Buckup 1993; Mago-Leccia 1994; Netto-Ferreira et al. 2009; Oyakawa and Mattox 2009; Caires and Figueiredo 2011; Peixoto et al. 2013) and diagnoses of species (e.g. Zanata et al. 2009; Marinho and Langeani 2010) as well as with the assistance of fish taxonomy experts. The use of the terms “cf”. “aff.”, and “sp”. follows Bengtson (1988). Taxonomic classification follows Reis et al. (2003). Voucher specimens are deposited in the Fish Collection of Universidade Federal do Oeste do Pará (UFOPA-I) (Appendix 1). Fish were collected under ICMBio license number 35649-2.

### Data analysis

An overall estimate of the fish species richness was calculated by means of the Jackknife 1 method (Krebs 1999), utilizing estimatS 8.2 (Cowel 2009). Alpha diversity was estimated by the Shannon-Wiener index (H’) (Shannon and Weaver 1963). To test difference in fish species composition among drainage systems, an analysis of similarities (ANOSIM) was applied with 999 permutations, using Bray-Curtis as a distance metric to measure the degree of dissimilarity between sites based on quantitative data (abundance) and Jaccard index for qualitative data (presence/absence of species). The analyses were done with the software PAST (Hammer et al. 2001).

## Results

**Figure 3.**
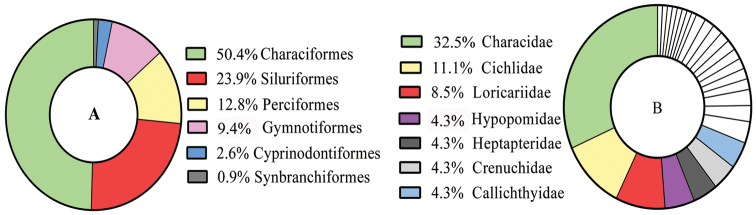
Representativeness of species for orders (**A**) and most diverse families (**B**) in streams of the Tapajós National Forest, Pará, Brazil.

A total of 3035 specimens belonging to 117 species, 27 families and six orders was sampled (Table 2; Appendix 2). The fish fauna was composed of 59 species of Characiformes (50.4%), 28 of Siluriformes (23.9%), 15 of Perciformes (12.8%), 11 of Gymnotiformes (9.4%), three of Cyprinodontiformes (2.6%) and one of Synbranchiformes (0.9%) (Fig. 3). The most representative families in number of species were Characidae with 38 species (32.5%), Cichlidae with 13 species (11.1%), and Loricariidae with ten species (8.5%) (Fig. 3).

**Table 2.** List of fish species collected in streams of the Tapajós National Forest, Pará, Brazil.

	DRAINAGE			
TAXON	Cupari	Curuá-Una	Tapajós	Total
**CHARACIFORMES**				
**Curimatidae**				
*Cyphocharax gangamon* Vari, 1992	-	-	17	17
*Cyphocharax gouldingi* Vari, 1992	8	-	-	8
**Anostomidae**				
*Leporinus granti* Eigenmann, 1912	1	-	-	1
*Leporinus friderici* (Bloch, 1794)	1	-	-	1
**Chilodontidae**				
*Chilodus punctatus* Müller & Troschel, 1844	8	-	-	8
**Crenuchidae**				
*Characidium* sp. 1	-	-	7	7
*Characidium* sp. 2	-	19	-	19
Characidium cf. zebra Eigenmann, 1909	41	-	-	41
*Elachocharax junki* (Géry, 1971)	-	-	38	38
*Crenuchus spilurus* Günther, 1863	-	-	20	20
**Gasteropelecidae**				
*Carnegiella strigata* (Günther, 1864)	1	-	-	1
**Characidae**				
*Astyanax bimaculatus* (Linnaeus, 1758)	9	-	-	9
Bryconops aff. caudomaculatus (Günther, 1864)	1	-	-	1
Bryconops cf. imitator Chernoff & Machado-Allison, 2002	36	-	-	36
Bryconops aff. melanurus (Bloch, 1794)	19	14	299	332
*Bryconops munduruku* Silva-Oliveira, Canto & Ribeiro, 2015	-	-	107	107
*Bryconops* sp.	1	-	-	1
*Creagrutus petilus* Vari & Harold, 2001	12	-	-	12
*Hemigrammus analis* Durbin, 1909	-	-	220	220
*Hemigrammus belottii* (Steindachner, 1882)	332	-	-	332
*Hemigrammus* sp.	-	-	13	13
*Hemigrammus levis* Durbin, 1908	-	-	1	1
*Hemigrammus hyanuary* Durbin, 1918	-	-	3	3
*Hemigrammus ocellifer* (Steindachner, 1882)	52	2	-	54
*Hemigrammus stictus* (Durbin, 1909)	-	-	1	1
*Hemigrammus vorderwinkleri* Géry, 1963	-	-	59	59
*Hyphessobrycon heterorhabdus* (Ulrey, 1894)	57	2	-	59
*Hyphessobrycon* sp. n.	-	16	-	16
Hyphessobrycon cf. agulha Fowler, 1913	-	-	2	2
*Iguanodectes variatus* Géry, 1993	-	-	25	25
*Jupiaba acanthogaster* (Eigenmann, 1911)	3	-	-	3
*Jupiaba apenima* Zanata, 1997	8	-	-	8
Jupiaba cf. potaroensis (Eigenmann, 1909)	105	-	-	105
*Jupiaba zonata* (Eigenmann, 1908)	2	-	-	2
Knodus cf. heteresthes (Eigenmann, 1908)	16	21	-	37
*Knodus* sp.	56	-	-	56
Knodus cf. shinahota Ferreira & Carvajal, 2007	-	2	-	2
*Microscemobrycon* sp.	1	-	-	1
*Moenkhausia celibela* Marinho & Langeani, 2010	-	-	6	6
*Moenkhausia collettii* (Steindachner, 1882)	7	-	-	7
*Moenkhausia hasemani* Eigenmann, 1917	4	-	-	4
*Moenkhausia comma* Eigenmann, 1908	5	6	3	14
*Moenkhausia oligolepis* (Günther, 1864)	54	-	-	54
*Moenkhausia* sp. n.	-	55	-	55
*Moenkhausia pirauba* Zanata, Birindelli & Moreira, 2009	4	-	-	4
*Moenkhausia* sp.	3	-	-	3
*Phenacogaster calverti* (Fowler, 1941)	96	-	-	96
*Phenacogaster* sp.	3	-	-	3
*Poptella compressa* (Günther, 1864)	13	-	-	13
**Serrasalmidae**				
*Catoprion mento* (Cuvier, 1819)	-	-	1	1
*Myloplus rubripinnis* (Müller &Troschel, 1844)	3	-	-	3
**Acestrorhynchidae**				
*Acestrorhynchus falcatus* (Bloch, 1794)	1	-	1	2
**Erythrinidae**				
*Erythrinus erythrinus* (Bloch & Schneider, 1801)	1	16	9	26
*Hoplias malabaricus* (Bloch, 1794)	12	2	3	17
*Hoplias curupira* Oyakawa & Mattox, 2009	-	1	-	1
**Lebiasinidae**				
*Copella nigrofasciata* (Meinken, 1952)	-	-	88	88
Pyrrhulina cf. brevis Steindachner, 1876	34	59	15	108
*Nannostomus eques* Steindachner, 1876	-	-	7	7
*Nannostomus* sp.	-	-	2	2
**SILURIFORMES**				
**Cetopsidae**				
*Denticetopsis seducta* Vari, Ferraris & de Pinna, 2005	1	-	-	1
*Denticetopsis* sp.	1	-	-	1
*Helogenes marmoratus* Günther, 1863	1	40	29	70
**Aspredinidae**				
*Bunocephalus coracoideus* (Cope, 1874)	-	1	-	1
*Bunocephalus knerii* Steindachner, 1882	1	-	-	1
**Trichomycteridae**				
*Ituglanis amazonicus* (Steindachner, 1882)	1	-	-	1
*Trichomycterus hasemani* (Eigenmann, 1914)	-	-	91	91
**Callichthyidae**				
*Aspidoras* sp. n.	2	-	-	2
*Callichthys callichthys* (Linnaeus, 1758)	1	1	-	2
Corydoras cf. approuaguensis Nijssen & Isbrücker, 1983	3	-	-	3
*Corydoras* sp.	4	-	-	4
*Megalechis picta* (Müller & Troschel, 1848)	-	-	1	1
**Loricariidae**				
*Ancistrus* sp.1	3	-	-	3
*Ancistrus* sp. 2 “bolinha”	1	-	-	1
*Curculionichthys* sp. n.	10	-	-	10
*Farlowella smithi* Fowler, 1913	3	-	-	3
*Farlowella* sp. 1 “juvenile”	1	-	-	1
*Farlowella* sp. 2	-	5	-	5
*Harttia dissidens* Rapp Py-Daniel & Oliveira, 2001	2	-	-	2
Hypostominae sp. “juvenile”	2	-	-	2
*Rineloricaria lanceolata* (Günther, 1868)	1	-	-	1
*Sturisoma* sp.	1	-	-	1
**Pseudopimelodidae**				
*Batrochoglanis raninus* (Valenciennes, 1840)	-	2	-	2
**Heptapteridae**				
*Brachyglanis microphthalmus* Bizerril, 1991	-	2	-	2
*Phenacorhamdia* sp.	6	-	-	6
*Pimelodella cristata* (Müller &Troschel, 1848)	2	-	-	2
*Pimelodella* sp.	5	-	-	5
*Rhamdia quelen* (Quoy & Gaimard, 1824)	1	2	-	3
**GYMNOTIFORMES**				
**Gymnotidae**				
*Gymnotus coatesi* La Monte, 1935	5	6	18	29
*Gymnotus coropinae* Hoedeman, 1962	11	15	1	27
**Sternopygidae**				
*Eigenmannia trilineata* López & Castello, 1966	-	4	-	4
*Sternopygus macrurus* (Bloch & Schneider, 1801)	-	3	-	3
**Rhamphichthyidae**				
*Gymnorhamphichthys petiti* Géry & Vu-Tân-Tuê, 1964	-	12	8	20
*Gymnorhamphichthys hypostomus* Ellis, 1912	1	-	-	1
**Hypopomidae**				
Brachyhypopomus aff. beebei (Schultz, 1944)	-	-	3	3
*Hypopygus lepturus* Hoedeman, 1962	6	51	14	71
*Hypopygus benoneae* Peixoto, Dutra, Santana & Wosiacki, 2013			2	2
Microsternarchus cf. bilineatus Fernández-Yépez, 1968	-	-	11	4
*Steatogenys duidae* (La Monte, 1929)	-	-	4	4
**CYPRINODONTIFORMES**				
**Rivulidae**				
*Rivulus urophthalmus* Günther, 1866	6	13	12	31
*Rivulus* sp.	-	-	6	6
**Poeciliidae**				
*Fluviphylax* sp.	-	-	3	3
**SYNBRANCHIFORMES**				
**Synbranchidae**				
*Synbranchus marmoratus* Bloch, 1795	3	10	7	20
**PERCIFORMES**				
**Polycentridae**				
*Monocirrhus polyacanthus* Heckel, 1840	-	-	2	2
**Cichlidae**				
*Aequidens* sp.	3	-	-	3
*Aequidens tetramerus* (Heckel, 1840)	25	91	7	123
*Acaronia nassa* (Heckel, 1840)	-	-	1	1
Apistogramma cf. agassizii (Steindachner, 1875)	-	-	154	154
*Apistogramma* sp. 1	1	33	1	35
*Apistogramma* sp. 2	-	-	4	4
*Crenicichla regani* Ploeg, 1989	-	-	14	14
*Crenicichla inpa* Ploeg, 1991	6	23	-	29
*Crenicichla pellegrini* Ploeg, 1991	-	-	1	1
*Dicrossus maculatus* Steindachner, 1875	-	-	4	4
*Hypselecara coryphaenoides* (Heckel, 1840)	-	-	1	1
*Satanoperca jurupari* (Heckel, 1840)	-	-	1	1
*Taeniacara candidi* Myers, 1935	-	-	3	3
**Gobiidae**				
*Microphilypnus acangaquara* Caires & Figueiredo, 2011	-	-	26	26
**TOTAL**	**1130**	**529**	**1376**	**3035**

The most abundant species were Bryconops aff. melanurus and *Hemigrammus belottii* (332 specimens each, 10.9% of the total species recorded), *Hemigrammus analis* (220 specimens, 7.2%), Apistogramma cf. agassizii (154 specimens, 5.1%), *Aequidens tetramerus* (123 specimens, 4.1%), Pyrrhulina cf. brevis (108 specimens, 3.6%), *Bryconops munduruku* (107 specimens, 3.5%), and Jupiaba aff. potaroensis (105 specimens, 3.5%). The abundances of these species together represented 48.8% of all collected specimens. Same species, despite the highest values of abundance, were restricted to one sampling station (e.g. *Hemigrammus analis* and *Trichomycterus hasemani*, collected at a single station, stream 21).The values of abundance, richness and diversity of the streams sampled are presented in Table 3.

**Table 3.** Values of abundance, richness and diversity (Shannon) of the sampled stations in streams in the Tapajós National Forest, Pará, Brazil.

STATION	ABUNDANCE	RICHNESS	DIVERSITY
**IG1**	87	14	2.11
**IG2**	23	8	1.67
**IG3**	422	21	1.61
**IG4**	59	9	1.83
**IG5**	39	10	1.93
**IG6**	63	10	1.82
**IG7**	125	21	2.53
**IG8**	99	23	2.53
**IG9**	438	30	2.43
**IG10**	148	14	1.99
**IG11**	82	10	1.37
**IG12**	108	8	0.56
**IG13**	0	0	0.00
**IG14**	51	15	1.51
**IG15**	403	8	0.81
**IG16**	78	15	2.36
**IG17**	13	7	1.73
**IG18**	0	0	0.00
**IG19**	24	5	1.28
**IG20**	64	10	1.69
**IG21**	566	28	1.91
**IG22**	142	11	2.03

**Figure 4.**
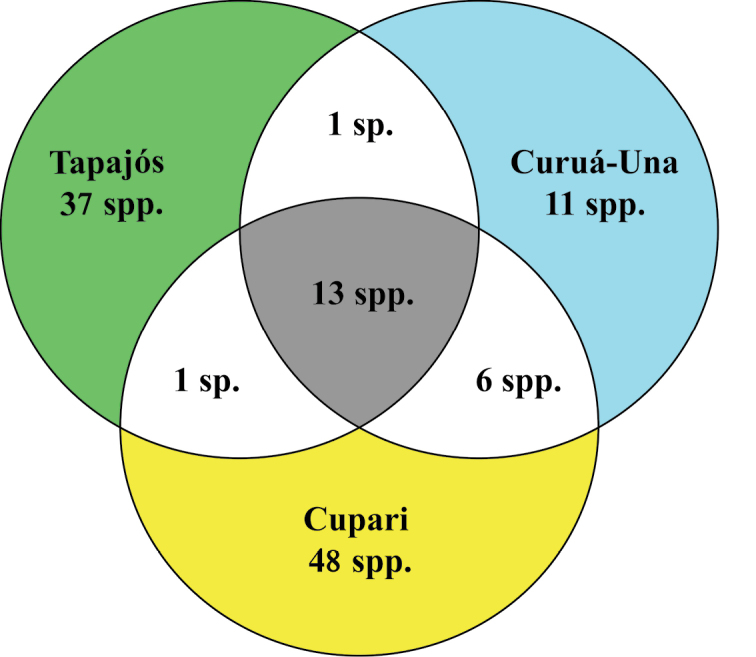
Distribution of fish species recorded in different drainage systems in the Tapajós National Forest.

The distribution of most species was related to drainage basins; from 117 species recorded, 38 were restricted to streams flow directly into the Tapajós River, 47 were collected only in streams draining into the Cupari River basin, and 11 were recorded only in streams draining into the Curuá-Una River basin. Six species were common to streams of the Curuá-Una and Cupari river drainages. One species was shared among streams flow directly into the Tapajós River and streams draining into the Curuá-Una River; thirteen species were shared among streams flow into the Curuá-Una and Cupari rivers, as well as streams draining directly into the Tapajós River (Fig. 4).

The analysis of similarities revealed a significant dissimilarity in fish species composition to both qualitative and quantitative data among drainage system in the Tapajos National Forest, as follows: Curuá-Una *vs.* Tapajós (presence/absence *R* = 0.32, p = 0.00; abundance *R* = 0.28, *p* = 0.01); Curuá-Una *vs.* Cupari (presence/absence *R* = 0.40, *p* = 0.01; abundance *R* = 0.36, *p* = 0.01); and Cupari *vs.* Tapajós (presence/absence *R* = 0.33, *p* = 0.02; abundance *R* = 0.23, *p* = 0.04).

## Discussion

The fish fauna of Tapajós National Forest, as well as the lower Tapajós River, is one of the most understudied and undersampled among aquatic systems in the Amazon drainage and so far all species found during the survey represent new records for the studied area. The number of species recorded (117) is one of the highest among known fish faunas in streams of the 1^st^ to 3^rd^ order in the Amazon drainage (e.g. Mendonça et al. 2005; Montag et al. 2008; De-Oliveira et al. 2009; Dias et al. 2009; Barros et al. 2011). However, the richness of species should be higher and reach up to 183 species. Therefore, more efforts should be employed in surveying the fish fauna of streams in the FLONA Tapajós.

The Neotropical fish faunas are characterized by the predominance of species from the orders Characiformes and Siluriformes (e.g. Angermeier and Karr 1983; Arbeláez 2004; Baumgartner et al. 2006; Arbeláez 2008; Scarabotti et al. 2011; Pedroza et al. 2012; Raiol et al. 2012; Claro-García and Shibatta 2013; Volcan et al. 2013, Ramos et al. 2014). Characiformes is one of the largest orders of fishes with at least 2000 valid species (Eschmeyer 2015). In the Neotropical region, Characiformes, Siluriformes, and Gymnotiformes, or Ostariophysi, constitute about 77% of the freshwater fish fauna; however the order Perciformes has over 515 freshwater species, in some cases alternating with Gymnotiformes as the third richest order (Albert et al. 2011). In the present study, Perciformes presented three species more than Gymnotiformes.

If families are concerned, the largest number of species in the Neotropical region is contained in Characidae and Loricariidae (Schaefer 1998); however, similar to this study, other faunistic surveys in small streams of the Amazon drainage revealed an inversion in the number of species in the families Cichlidae and Loricariidae (e.g. Mendonça et al. 2005; Barros et al. 2011).

The highest values of richness were observed at sampling stations 8, 9 and 21 (Table 3). Stations 8 and 9 were at river sections characterized by the greatest depth and width. In streams, studies indicate that an increase in species richness is positively related to the habitat complexity and shelter availability as well as current velocity and stream size (Garutti 1988; Meffe and Sheldon 1988; Abes and Agostinho 2001; Súarez and Lima-Junior 2009). In the Neotropical region, substrate, depth and current speed are among the most important physical features, and a combination of such environmental features produces a mosaic of microhabitats, which can explain the downstream increase in species richness (Casatti 2005).

Station 21 is near to the mouth of a stream draining into a lake, and its high values of richness is resulted of the presence of species typically recorded near lakes such as *Catoprion mento*, *Hemigrammus analis*, *Hemigrammus levis*, *Hemigrammus hyanuary*, *Hemigrammus stictus* and *Dicrossus maculatus* (Siqueira-Souza and Freitas 2004; Lima et al. 2013; Kullander 2011). Four new species were recorded, *Curculionichthys* sp. n., *Aspidoras* sp. n., *Hyphessobrycon* sp. n., and *Moenkhausia* sp. n, the *Aspidoras* being known only from the present study. Some specimens received provisional identification with the use of “cf. “, “aff.”, or “sp.”, which may be indicative of the recognition of other new species after more refined analysis, or may even indicate insufficient research for some taxonomic groups (e.g. *Ancistrus* and *Apistogramma*).

The existence of dissimilarity in fish species composition of different, however geographically close, drainage systems within the Tapajos National Forest indicates that geographic isolation coupled with environmental characteristics is responsible for the structuring of fish communities, in accordance with observed by Schleuter et al. (2012) in temperate regions and Barros et al. 2013, in tropical streams. Furthermore the smaller drainage basins can significantly influence the stream fish assemblages composition (Mendonça et al. 2005; Barros et al. 2013) since headwaters streams often support exclusive species that do not occur in the river system, allowing constitute single assemblages that are fundamental to compose the regional fish diversity (Paller 1994; Meyer et al. 2007) and must be priority included in units conservation planning for freshwater systems.

## Authors’ contribution statement

CSO, ACC and FRR collected the data, identified the species, filled the database and wrote the text.

## Figures and Tables

**Figure:**
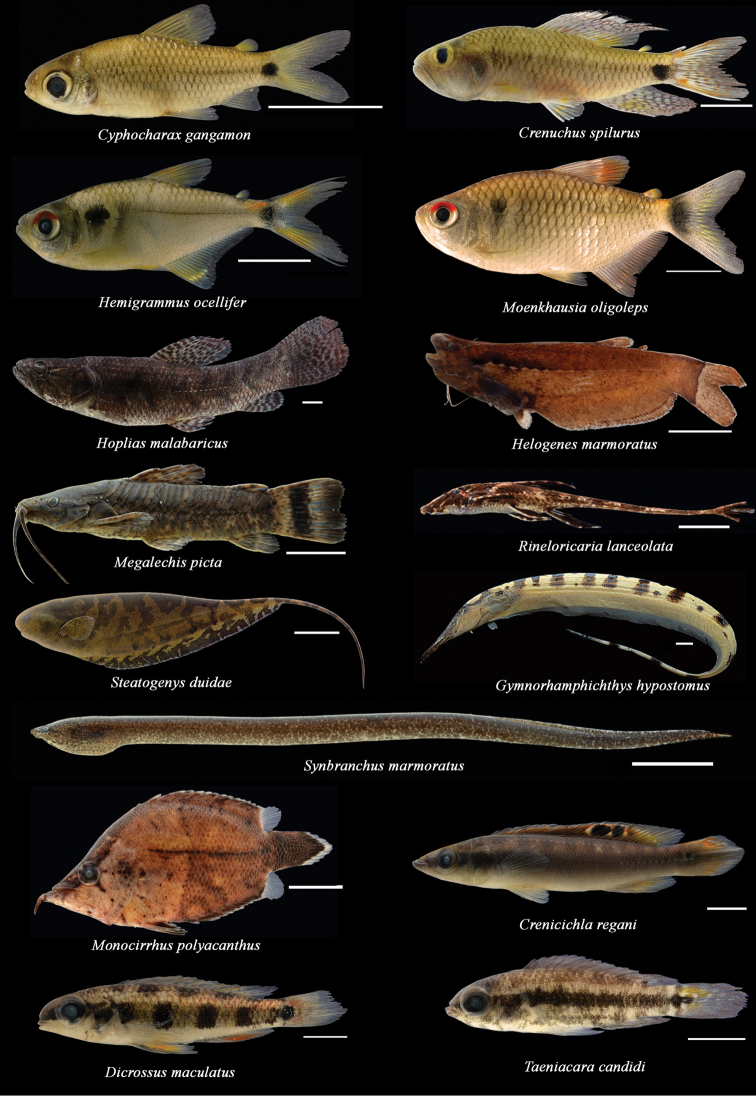
Some fish species collected in the streams of the Tapajós National Forest, Pará, Brazil. Scale bar 1 cm.

## Appendix 1

Voucher specimens.

**CHARACIFORMES**: *Cyphocharax gangamon* (UFOPA-I 00574), *Cyphocharax gouldingi* (UFOPA-I 00459); *Leporinus granti* (UFOPA-I 00444); *Leporinus friderici* (UFOPA-I 00536); *Chilodus punctatus* (UFOPA-I 00455); *Characidium* sp.1 (UFOPA-I 00366); *Characidium* sp. 2 (UFOPA-I 00360, 00424); Characidium cf. zebra (UFOPA-I 00435, 00454, 00530); *Elachocharax junki* (UFOPA-I 00370); *Crenuchus spilurus* (UFOPA-I 00369, 00595); *Carnegiella strigata* (UFOPA-I 00529); *Astyanax bimaculatus* (UFOPA-I 00511, 00554); Bryconops aff. caudomaculatus (UFOPA-I 00431); Bryconops aff. melanurus (UFOPA-I 00365, 00384, 00402, 00432, 00494, 00571); *Bryconops munduruku* (UFOPA-I 00495, 00504); Bryconop cf. imitator (UFOPA-I 00453, 00606) *Bryconops* sp. (UFOPA-I 00433); *Creagrutus petilus* (UFOPA-I 00437, 00458); *Hemigrammus analis* (UFOPA-I 00577); *Hemigrammus belottii* (UFOPA-I 00521, 00532); *Hemigrammus* sp. (UFOPA-I 00498); *Hemigrammus levis* (UFOPA-I 00579); *Hemigrammus hyanuary* (UFOPA-I 00578); *Hemigrammus ocellifer* (UFOPA-I 00346, 00522, 00533, 00545, 00557); *Hemigrammus stictus* (UFOPA-I 00580); *Hemigrammus vorderwinkleri* (UFOPA-I 00373, 00599), *Hyphessobrycon heterorhabdus* (UFOPA-I 00419, 00464); *Hyphessobrycon* sp. n. (UFOPA-I 00347, 00359, 00407, 00420); Hyphessobrycon cf. agulha (UFOPA-I 00500); *Iguanodectes variatus* (UFOPA-I 00377, 00389); *Jupiaba acanthogaster* (UFOPA-I 00468); *Jupiaba apenina* (UFOPA-I 00467); Jupiaba cf. potaroensis (UFOPA-I 00441, 00466); *Jupiaba zonata* (UFOPA-I 00442); *Knodus heteresthes* (UFOPA-I 00395, 00408, 00422, 00443); *Knodus* sp. (UFOPA-I 00514, 00559); Knodus cf. shinahota (UFOPA-I 00423); *Microscemobrycon* sp. (UFOPA-I 00470); *Moenkhausia celibela* (UFOPA-I 00584); *Moenkhausia collettii* (UFOPA-I 00537); Moenkhausia cf. hasemani (UFOPA-I 00445; 00515); *Moenkhausia comma* (UFOPA-I 00350, 00397, 00426, 00490, 00501, 00509, 00560); *Moenkhausia oligolepis* (UFOPA-I 00446, 00472, 00516, 00538); *Moenkhausia* sp. n. (UFOPA-I 00349, 00396, 00409, 00425, 00489); *Moenkhausia pirauba* (UFOPA-I 00471); *Moenkhausia* sp. (UFOPA-I 00539); *Phenacogaster calverti* (UFOPA-I 00474); *Phenacogaster* sp. (UFOPA-I 00475); *Poptella compressa* (UFOPA-I 00477); *Catoprion mento* (UFOPA-I 00572); *Myloplus rubripinnis* (UFOPA-I 00473); *Acestrorhynchus falcatus* (UFOPA-I 00363, 00451); *Erythrinus erythrinus* (UFOPA-I 00344, 00356, 00386, 00404, 00404, 00404, 00415, 00483, 00555, 00596, 00603); *Hoplias malabaricus* (UFOPA-I 00374, 00418, 00463, 00499, 00513, 00523, 00534); *Hoplias curupira* (UFOPA-I 00604); *Copella nigrofasciata* (UFOPA-I 00367, 00385, 00505, 00594); Pyrrhulina cf. brevis (UFOPA-I 00398, 00491, 00510, 00524, 00540, 00546, 00551, 00561, 00588); *Nannostomus eques* (UFOPA-I 00586); *Nannostomus* sp. (UFOPA-I 00587); **SILURIFORMES**: *Denticetopsis seducta* (UFOPA-I 00414); *Denticetopsis* sp. (UFOPA-I 00355); *Helogenes marmoratus* (UFOPA-I 00341, 00358, 00362, 00383, 00394, 00406, 00417, 00487, 00497, 00508, 00542, 00549); *Bunocephalus coracoideus* (UFOPA-I 00343); *Bunocephalus knerii* (UFOPA-I 00434); *Ituglanis amazonicus* (UFOPA-I 00558); *Trichomycterus hasemani* (UFOPA-I 00592); *Aspidoras* sp. n. (UFOPA-I 00430), *Callichthys callichthys* (UFOPA-I 00512, 00543); Corydoras cf. approuaguensis (UFOPA-I-00436, 00456); *Corydoras* sp. (UFOPA-I 00457); *Megalechis picta* (UFOPA-I 00581); *Ancistrus* sp.1 (UFOPA-I 00429); *Ancistrus* sp.2 (UFOPA-I 00452); *Farlowella smithi* (UFOPA-I 00438, 00460); *Farlowella* sp. 1 “juvenile” (UFOPA-I 00461); *Farlowella* sp. 2 (UFOPA-I-00605); Hypostominae sp. “juvenile” (UFOPA-I 00465); *Harttia dissidens* (UFOPA-I 00469); *Curculionichthys* sp. n. (UFOPA-I 00479); *Rineloricaria lanceolata* (UFOPA-I 00606); *Sturisoma* sp. (UFOPA-I 00448); *Batrochoglanis raninus* (UFOPA-I 00411); *Brachyglanis microphthalmus* (UFOPA-I 00401, 00412); *Phenacorhamdia* sp. (UFOPA-I 00439, 00462); *Pimelodella cristata* (UFOPA-I 00447); *Pimelodella* sp. (UFOPA-I 00476); *Rhamdia quelen* (UFOPA-I 00351, 00517); **GYMNOTIFORMES**: *Gymnotus coatesi* (UFOPA-I 00372, 00388, 00485, 00507, 00531, 00597); *Gymnotus coropinae* (UFOPA-I 00345, 00486, 00496, 00520, 00550, 00556); *Eigenmannia trilineata* (UFOPA-I 00393); *Sternopygus macrurus* (UFOPA-I 00428); *Gymnorhamphichthys petiti* (UFOPA-I 00357, 00405, 00416, 00484); *Gymnorhamphichthys hypostomus* (UFOPA-I 00440); Brachyhypopomus aff. beebei (UFOPA-I 00569); *Hypopygus lepturus* (UFOPA-I 00348, 00375, 00421, 00488, 00535); *Hypopygus benoneae* (UFOPA-I 00381); *Microsternarchus bilineatus* (UFOPA-I 00378, 00570)); *Steatogenys duidae* (UFOPA-I 00380, 00601); **CYPRINODONTIFORMES**: *Rivulus urophthalmus* (UFOPA-I 00379, 00390, 00399, 00427, 00502, 00518, 00547, 00552); *Rivulus* sp. (UFOPA-I 00589, 00600); *Fluviphylax* sp. (UFOPA-I 00576); **SYNBRANCHIFORMES**: *Synbranchus marmoratus* (UFOPA-I 00352, 00478, 00492, 00525, 00562, 00591, 00602); **PERCIFORMES**: *Monocirrhus polyacanthus* (UFOPA-I 00585); *Aequidens* sp. (UFOPA-I 00541); *Aequidens tetramerus* (UFOPA-I 00339, 00353, 00361, 00382, 00391, 00400, 00410, 00449, 00480, 00481, 00503, 00519, 00526, 00548, 00553, 00563); *Acaronia nassa* (UFOPA-I 00564); Apistogramma cf. agassizii (UFOPA-I 00364, 00493, 00565, 00593); *Apistogramma* sp. 1 (UFOPA-I 00342, 00528, 00567); *Apistogramma* sp. 2 (UFOPA-I 00568); *Crenicichla inpa* (UFOPA-I 00340, 00354, 00392, 00403, 00413, 00450, 00482, 00527, 00544); *Crenicichla pellegrini* (UFOPA-I 00506); *Crenicichla regani* (UFOPA-I 00368, 00573); *Dicrossus maculatus* (UFOPA-I 00575); *Hypselecara coryphaenoides* (UFOPA-I 00376); *Satanoperca jurupari* (UFOPA-I 00590); *Taeniacara candidi* (UFOPA-I 00566); *Microphilypnus acangaquara* (UFOPA-I 00582, 00583).
